# Extremely high levels of central nervous system involvement in miliary tuberculosis

**DOI:** 10.1186/s12879-022-07390-7

**Published:** 2022-04-29

**Authors:** Guirong Wang, Ruixia Liang, Qing Sun, Xinlei Liao, Chenqian Wang, Hairong Huang

**Affiliations:** 1 National Clinical Laboratory on Tuberculosis, Beijing Key Laboratory on Drug-Resistant Tuberculosis, Beijing Chest Hospital, Capital Medical University, Beijing Tuberculosis and Thoracic Tumor Research Institute, Beiguan St. #9, Beijing, 101149 China; 2 Tuberculosis Department, Henan Chest Hospital, Zhengzhou, 450001 China

**Keywords:** Central nervous system, Cerebrospinal fluid, Miliary, Tuberculosis

## Abstract

**Background:**

Miliary tuberculosis (TB) is one of the severest manifestations of TB that can be lethal when concomitant with the central nervous system (CNS) involvement. Bacteriological, biochemical and radiological methods for find CNS comorbidity in miliary TB was evaluated in this study.

**Methods:**

Consecutive miliary TB adults were retrospectively enrolled from two designated TB hospitals in China. The capacities of examinations of cerebrospinal fluid (CSF), cerebral computed tomography (CT) and magnetic resonance imaging (MRI) for diagnosis of CNS involvement were assessed.

**Results:**

Assessment of CNS involvement with a lumbar puncture and/or neuroimaging was undertaken in 282 out of 392 of acute miliary TB. Of these 282 patients, 87.59% (247/282) had CNS involvement. Cerebral contrast-enhanced MRI (96.05%, 170/177) and MRI (93.15%, 204/219) yielded significantly higher sensitivities over CSF examination (71.92%, 146/203, P < 0.001) and CT (34.69%, 17/49, P < 0.001). The sensitivity of CSF examination was superior to CT scan (P < 0.001). Although 59.65% (134/225) miliary TB patients acquired bacteriological evidence with sputum examination, the positivity was only 8.82% (21/238) for CSF examination by conventional and molecular tests.

**Conclusion:**

Almost all miliary TB had CNS involvement and MRI demonstrated outstanding potential over other methods. Therefore, a routinely screening of CNS TB should be strongly suggested in miliary TB and MRI could be used as the initial approach in resources rich settings.

## Background

Miliary tuberculosis (TB) is caused by the acute, diffused dissemination of *Mycobacterium tuberculosis* (Mtb) from the primary site of infection, generally as a consequence of inadequate host defenses [1, 2]. Approximately, 61% of the reported miliary TB have complications of extrapulmonary involvement [3]. Central nervous system (CNS) TB presents the most severe form and causes substantial morbidity and mortality [4]. Great challenges exist in diagnosis and management of CNS-TB: rapid, sensitive, and affordable diagnostic tests remain beyond available; an appropriate management algorithm is yet to be established [5].

In order to diagnose CNS involvement, detection of acid-fast bacilli by smear or/and culture of cerebrospinal fluid (CSF) are routinely performed, despite the disappointing sensitivities of these two methods [6]. In 2013, the WHO endorsed the Xpert MTB/RIF assay (Cepheid, Sunnyvale, CA, USA) as the preferred initial test for tuberculous meningitis (TBM) [7]. Due to its imperfect sensitivity, the Xpert assay cannot be used as a rule-out test for TBM [8]. The biochemistry testing of CSF was another frequently used approach for diagnosing CNS-TB with mild sensitivity but low specificity [9].

Furthermore, the delayed occurrence of CSF abnormality was another concern. The neuroimaging with computed tomography (CT) and magnetic resonance imaging (MRI) was used more frequently due to its high sensitivity, rapid and none invasive characterizations, but higher costs limited their usage in poor settings, despite the fact that they harbor majority of the CNS-TB. Up to now, diagnosis of CNS involvement relies on combining supportive clinical, laboratory, and radiological findings [10].

CNS involvement has been described in 10–30% of adult patients with miliary TB before the modern radiological technique was applied [11, 12], whereas limited data have been published after the more sensitive approaches for diagnosis of CNS involvement are being used widely. In this retrospective study, we evaluated the capacities of bacteriological and biochemical examinations of CSF, CT, and MRI for diagnosis of CNS involvement among miliary TB in a high TB burden country (58 new TB cases per 100,000 population per year). Similar study has never been performed before.

## Patients and methods

### Study population

The study was conducted in two TB designated hospitals: Beijing Chest Hospital (Beijing, China) and Henan Chest Hospital (Zhengzhou, Henan province, China). All adult patients (aged 15 years or older) hospitalized with miliary TB between June 2012 and April 2017 in Beijing Chest Hospital, between January 2016 and July 2017 in Henan Chest Hospital were identified from the computerized databases. The medical records were investigated for demographic characteristics, clinical features, and different investigations results. Assessment of CNS involvement with a lumbar puncture and/or neuroimaging was undertaken. CSF was subjected to routine biochemistry examination, smear microscopy, culture, and nucleic acid amplification tests. Cerebral CT, contrast-enhanced CT, MRI or contrast-enhanced MRI was performed. HIV testing using chemiluminescence microparticle immuno assay was done for all the patients. If the patient was HIV-positive, he would be transferred to HIV designated hospital for health care according to our administrative police. Therefore, all the enrolled patients were HIV-uninfected in this study.

### Criteria for miliary TB diagnosis

Presence of miliary pattern on chest radiograph with or without evidence of multi-organ involvement, along with one or more of the following features [1]: (1) clinical features compatible with TB, including cough for 3 weeks or more, fever, weight loss, night sweats, loss of appetite or haemoptysis, and responding to antituberculosis treatment; (2) positive smear or culture for TB; and (3) histopathological evidence of TB.

### Criteria for CNS involvement diagnosis among miliary TB

The miliary TB who had one or more of the following features was diagnosed as having CNS involvement: (1) neuroimaging abnormal presentations (hydrocephalus, basal meningeal enhancement, infarcts, tuberculoma, and pre-contrast basal hyperdensity); (2) bacteriological evidence of TB with CSF examination by conventional and/or molecular tests; (3) had likely neural symptoms (one or more of the following: headache, irritability, vomiting, fever, neck stiffness, convulsions, focal neurological deficits, or altered consciousness) with supportive CSF biochemistry examination outcomes [a lymphocytic pleocytosis with cells 10–500/mm^3^ (> 50% lymphocytes), moderately to severe elevated protein content (0.5–3.0 g/l) and glucose levels lower than 45 mg/dl or below 40–50% of serum glucose].

### Statistical analyses

The statistical analysis was conducted using SPSS (version 19.0 software, IBM, Armonk, NY, USA). The chi-squared test was used for statistical analysis and a P value of < 0.05 was considered statistically significant.

## Results

### Patient enrollment

A total of 14,946 pulmonary TB patients have been documented in the two hospitals, 392 (2.62%) of them had acute miliary TB. 282 (71.94%) miliary TB patients undertook lumbar punctures or/and neuroimaging for the assessment of CNS involvement. Of these 282 patients, 87.59% (247/282) had CNS involvement. Totally, 8.50% (21/247) patients were definite cases by detection of Mtb in CSF, among which 19 were in CSF abnormal group and 2 in CSF normal group. The other 226 enrolled patients had clinical diagnosis.

### Demographic characteristics and clinical features

Among the 392 miliary TB, the male to female ratio was 1.24. The median age was 36 years (range 15–87), and 18–39 years group accounted for 53.06% of all enrolled patients. The most frequent symptom of TB was fever (90.56%), followed by cough (46.68%). The most common symptoms suggestive of CNS-TB were headache (40.31%), vomiting (25.00%) and meningeal signs (17.09%). All the patients were HIV-uninfected (Table 1). Furthermore, Table 2 showed the sites of extrapulmonary TB other than CNS in the enrolled miliary TB patients, e.g. 70 (17.86%) cases with osteoarticular involvement, and 59 (15.05%) with pleural involvement.

**Table 1:** Demographic and clinical characteristics of 392 individuals with acute miliary tuberculosis

Characteristics	Overall	Undergo investigations for CNS TB	Without investigations for CNS TB	P values
Total	392	282	110	
Sex				0.167
Male	217 (55.36)	150 (53.19)	67 (60.91)	
Female	175 (44.64)	132 (46.81)	43 (39.09)	
Age, median (range), year	36 (15–87)	27 (15–83)	42 (16–87)	< 0.001
Age categories years				
15–18	18 (4.59)	14 (4.96)	4 (3.64)	
18–39	208 (53.06)	161 (57.09)	47 (42.73)	
40–59	74 (18.88)	53 (18.79)	21 (19.09)	
> 60	92 (23.47)	54 (19.15)	38 (34.55)	
HIV status				–
Negative	392 (100.00)	282 (100.00)	110 (100.00)	
Positive	0	0	0	
Presenting symptoms				
Fever	355 (90.56)	259 (91.84)	96 (87.27)	0.164
Cough	183 (46.68)	137 (48.58)	46 (41.82)	0.228
Weight loss	149 (38.01)	107 (37.94)	42 (38.18)	0.965
Headache	158 (40.31)	147 (52.13)	11 (10.00)	< 0.001
Vomiting	98 (25.00)	97 (34.40)	1 (0.91)	< 0.001
Confusion	61 (15.56)	48 (17.02)	13 (11.82)	0.202
Altered consciousness	31 (7.91)	20 (7.09)	11 (10.00)	0.338
Convulsion	12 (3.06)	9 (3.19)	3 (2.73)	0.811
Seizures	8 (2.04)	8 (2.84)	0 (0.00)	0.074
Meningeal sign	67 (17.09)	60 (21.28)	7 (6.36)	< 0.001
Cranial nerve palsy	42 (10.71)	36 (12.77)	6 (5.45)	0.035
Coma	16 (4.08)	16 (5.67)	0 (0.00)	0.011
Paraplegia	9 (2.30)	6 (2.13)	3 (2.73)	0.722

**Table 2:** The sites of extrapulmonary tuberculosis other than the CNS in 392 patients with miliary tuberculosis

Extrapulmonary tuberculosis	n(%)
Osteoarticular tuberculosis	70 (17.86)
Pleural tuberculosis	59 (15.05)
Lymphatic tuberculosis	22 (5.61)
Peritoneal tuberculosis	12 (3.06)
Genitourinary tuberculosis	11 (2.81)
Intestinal tuberculosis	10 (2.55)
Splenic tuberculosis	9 (2.30)
Tracheobronchial tuberculosis	3 (0.77)
Laryngeal tuberculosis	3 (0.77)
Choroidal tuberculosis	2 (0.51)

### Bacteriological findings

In total, 317 miliary TB were subjected to at least one kind of bacteriological test with sputum and/or blood and/or CSF. 151 (47.63%) of them produced positive outcomes by either tests (Table 3). Xpert MTB/RIF (Xpert) (Cepheid, Sunnyvale, USA) acquired highest positivity with sputum, followed by conventional PCR (Daan gene Ltd., Guangzhou, China), culture and MeltPro TB (Zeesan Biotech, Xiamen, China). Altogether, 59.65% (134/225) of miliary TB demonstrated bacteriological evidence of TB. CSF testing yielded much lower positivity compared with sputum for all the methods performed. Only 11.63% (15/129) of CSF were positive for Xpert, whereas the other tests were even lower. As a consequence, only 8.82% of the miliary TB had definite CNS-TB.

**Table 3:** Microbiological findings for miliary tuberculosis and cerebrospinal fluid examination

Specimen	Smear (%)	Culture (%)	Xpert (%)	PCR (%)	MeltPro TB (%)	Total
Sputum	47/179 (26.26)	80/161 (49.69)	67/109 (61.47)	37/70 (52.86)	30/66 (45.45)	134/225 (59.56)
Blood	–	4/32 (12.50)	–	–	–	4/32 (12.50)
CSF	2/206 (0.97)	6/87 (6.90)	15/129 (11.63)	9/188 (4.79)	–	21/238 (8.82)
Total	49/287 (17.07)	89/219 (40.64)	77/166 (46.39)	46/218 (21.10)	30/66 (45.45)	151/317 (47.63)

### CSF biochemistry test findings

Assessment of CNS involvement with a lumbar puncture was undertaken in 60.71% (238/392) miliary TB patients. CSF abnormalities were detected in 146 patients overall. Headache, vomiting, confusion, altered consciousness, meningeal sign and cranial nerve palsy were noted in a much higher proportion among CSF abnormal patients than among CSF normal cases (Table 4).

**Table 4:** The presenting symptoms in 238 miliary tuberculosis with lumbar puncture

Methods	CSF normal (n = 92)			CSF abnormal (n = 146)	P values*
	With CNS diagnosis (n = 57)	Without CNS diagnosis (n = 35)	Total		
Fever	32 (91.43%)	50 (87.72%)	82 (89.13%)	140 (95.89%)	0.043
Cough	20 (57.14%)	28 (49.12%)	48 (52.17%)	57 (39.04%)	0.047
Weight loss	15 (42.86%)	23 (40.35%)	38 (44.19%)	61 (41.78%)	0.942
Headache	6 (17.14%)	20 (35.09%)	26 (28.26%)	108 (73.97%)	< 0.001
Vomiting	3 (8.57%)	10 (17.54%)	13 (14.13%)	66 (45.21%)	< 0.001
Confusion	0	2 (3.51%)	2 (2.33%)	42 (28.77%)	< 0.001
Altered consciousness	0	5 (8.77%)	5 (5.81%)	22 (15.07%)	0.022
Convulsion	0	3 (5.26%)	3 (3.49%)	6 (4.11%)	0.738
Seizures	0	1 (1.75%)	1 (1.16%)	2 (1.44%)	0.849
Meningeal sign	1 (2.86%)	5 (8.77%)	6 (6.52%)	45 (30.82%)	< 0.001
Cranial nerve palsy	2 (5.71%)	2 (3.51%)	4 (4.65%)	21 (14.38%)	0.014
Coma	0	2 (3.51%)	2 (2.33%)	10 (6.85%)	0.108
Paraplegia	0	0	0	8 (5.48%)	0.022

*CSF* cerebrospinal fluid, *CNS* central nervous system

There was no significant difference between CSF normal patients with or without CNS involvement groups

^*^Comparison between CSF normal and abnormal groups

### Radiographic image findings

Among 223 miliary TB, neuroimaging examinations, cerebral CT, contrast-enhanced CT, MRI and contrast-enhanced MRI were performed for 49, 8, 219 and 177 patients, respectively. 31 patients underwent 1 neurological imaging and 26 with CSF examination; 164 patients underwent 2 neurological imagings and 128 with CSF examination; 27 patients underwent 3 neurological imagings and 25 with CSF examination; 1 patients underwent 4 neurological imagings and with CSF examination. The most common abnormality on neuroimaging was the presence of miliary pattern of brain, followed by meningeal enhancement (Table 5).

**Table 5:** Radiographic findings of 247 miliary tuberculosis patients with central nervous system involvement

Characteristics	CT (n = 49, %)	Contrast-enhanced CT (n = 8, %)	MRI (n = 219, %)	Contrast-enhanced MRI (n = 177, %)
Meningeal TB				
Meningeal enhancement	4 (8.16)	1 (12.50)	39 (17.81)	50 (28.25)
Meningeal tuberculomas	0	0	8 (3.76)	19 (10.73)
Meningeal abscess	0	0	1 (0.46)	2 (1.13)
Brain parenchymal TB				
Miliary pattern of brain	17 (34.69)	7 (87.50)	184 (84.02)	157 (88.70)
Tuberculous encephalitis	0	0	6 (2.74)	1 (0.46)
Mixed intracranial TB	3 (6.12)	0	19 (8.68)	37 (20.90)

### Overall sensitivity for identification CNS involvement among miliary TB

Among the 247 miliary TB with CNS involvement, contrast-enhanced MRI (96.05%, 170/177) and MRI (93.15%, 204/219) were significantly more sensitive than CSF examination (71.92%, 146/203, P < 0.001) and CT (34.69%, 17/49, P < 0.001). The sensitivity of CSF examination was superior to CT scan (P < 0.001). Contrast-enhanced MRI had higher sensitivity than MRI, but the differences were not significant (P = 0.211) (Table 6). Among patients with or without CNS symptom, MRI and contrast-enhanced MRI had more than 90% abnormal manifestations, whereas the abnormal rates for CT were much lower for both groups (Table 5). Surprisingly, 5 out of 7 patients without CNS symptom had abnormal CT imaging, whereas only 12 out of 42 patients with CNS symptom had abnormal CT imaging. However, the patient number for non-CNS symptom group was too small to draw any conclusion.

**Table 6:** The abnormal rate of cerebrospinal fluid or radiographic imaging stratified by central nervous system symptoms for miliary tuberculosis patients with central nervous system involvement

Methods	Total	Without CNS symptoms (n = 73)	With CNS symptoms (n = 174)	P values
CSF	146/203 (71.92)	24/56 (42.86)	122/147 (82.99)	< 0.001
CT	17/49 (34.69)	5/7 (71.43)	12/42 (28.57)	0.027
MRI	204/219 (93.15)	67/70 (94.03)	137/149 (91.95)	0.303
Contrast-enhanced MRI	170/177 (96.05)	55/57 (96.43)	115/120 (95.80)	0.834

Among the 146 CSF abnormal CNS-TB, 28.5% (10/35) of the patients had cerebral CT scanning which presented abnormal images, while these percentages for contrast-enhanced CT, MRI and contrast-enhanced MRI were 80% (4/5), 92.00% (115/125), and 95.00% (95/100), respectively. In addition, among the 57 CNS TB with normal CSF outcomes, CT, contrast-enhanced CT, MRI and contrast-enhanced MRI detected 36.36% (4/11), 100% (3/3), 98.04% (50/51), 100% (38/38) patients. Notably, among 8 patients with CNS symptom but with normal CT images and normal CSF outcomes, lesions in brains were identified in 4 patients using MRI and contrast-enhanced MRI. Another patient, using contrast-enhanced CT, also showed cerebral abnormality.

Stratification analysis was conducted further by dividing the miliary TB into definite or clinically diagnosed CNS-TB groups. MRI and contrast-enhanced MRI demonstrated similar sensitivities among both groups, the positive rates were all greater than 90%, and no significant difference was observed. CSF examination had higher sensitivity for the definite CNS-TB group (90.48%, 19/21) than for clinically diagnosed group (69.78%, 127/182; P = 0.046). Whereas CT scan had lower sensitivities for both groups (50.00%, 2/4 vs 33.33%, 15/45; P = 0.502), and no significant difference was observed.

## Discussion

Along with the advancement of new techniques increasing number of miliary TB with CNS comorbidity was identified [13–15], but systematic evaluation of different methods to find CNS-TB has never been performed before. In this study, we first compared all the available approaches for diagnosis of CNS-TB in a setting with enough facilities and capacity for TB diagnosis. As an invasive method, lumbar puncture has been proved to be less optimal. 38.66% (92/238) of patients subjected to lumbar punctuation acquired normal CSF outcomes, whereas 91.23% (52/57) of them had abnormal neuroimaging. In contrast with CT, MRI presented obvious advantage for CNS-TB diagnosis, which is consistent with other reports [6, 13, 15]. Even among miliary TB with no CNS symptom, 94.03% (63/67) and 96.43% (54/56) produced abnormal images by MRI or contrast-enhanced MRI, whereas the rate for CT and CSF examination were 71.43% (5/7) and 42.86% (24/56), respectively. Botha H and colleagues [16] reported that a normal CT scan was not uncommon in early tuberculous meningitis (TBM). A study from India reported that among 48 TBM, only 30 patients (62.5%) presented abnormalities on contrast-enhanced CT [15]. Gupta et al. [17] performed contrast MRI on 7 miliary TB with no CNS symptom, and maging revealed that all patients had asymptomatic brain involvement and a gradual resolution of lesions was noted on follow-up. The most important finding in our study was that almost all miliary TB who undertook tests for CNS involvement had CNS-TB (87.59%). This rate is much higher than previous reports [17, 18]. This could be because we included all the available approaches for CNS-TB diagnosis, especially the highly sensitive MRI and contrast-enhanced MRI. It is reasonable to presume that miliary TB who did not undertake any test for CNS involvement screening, or who only had a less sensitive CSF examination, could also have lesions in CNS. Additionally, the emergence of more sensitive methods than MRI in the future is possible, which could lead to diagnosis of an even higher percentage of CNS involvement in miliary TB. Based on the outcomes in this assay, for all the miliary TB, a routinely screening of CNS involvement should be strongly suggested.

Not only the suboptimal sensitivity, but also the delayed change of CSF examination is a major concern. Majority of patients with abnormal neuroimaging did not present CSF abnormality. Additionally, two cases of culture-proven TBM with no other CSF abnormalities were identified in this study, which has also been reported in another study [13]. The emerging of CNS symptom during the treatment targeting pulmonary TB highlighted the differences in treatment strategy between pulmonary TB and CNS-TB and stressed the importance to identify the CNS involvement even if the patient did not show symptoms.

As always, CSF bacteriological examination had low yields. Although molecular tests, including Xpert, gained extra sensitivity in contrast with the conventional tests, in general, all the methods had very low sensitivities with the paucibacillary specimen [19]. This fact lowers the value of bacteriological examination even though it is the only way to pursue evidence for definite diagnosis of CNS-TB. A recent publication reported that a next generation Xpert assay- Xpert Ultra could increase the sensitivity of CSF detection evidently [20–22], and new technology like that is urgently needed. In contrast to the CSF examination, sputum examination with all the methods had much better sensitivities. 59.56% of the enrolled miliary TB acquired bacteriological evidence, which could support the CNS-TB diagnosis to a great extent as well. However, only two third of the enrolled miliary TB undertook sputum examination in this assay, the other patients did not do the test because they could not expectorate sputum. According to our assay, sputum bacilli detection is of great value for miliary TB diagnosis and CNS-TB diagnosis, so educating patient to collect sputum and high-osmosis sodium sputum induction are of importance.

A clear diagnosis of CNS involvement in miliary TB will incur ancillary treatment with cortisone, elongated treatment course, and drug adjustment with better blood–brain barrier permeation efficiency. Adjunct corticosteroid treatment is commonly administered for CNS TB, while less frequently for miliary TB without CNS involvement [23]. In the absence of associated meningeal involvement, the guidelines enacted by the American Thoracic Society, the Centers for Disease Control and Prevention of America, the Infectious Disease Society of America, and the British Thoracic Society state that 6 months of treatment will be administered to miliary TB; in the presence of associated TBM, treatment needs to be given for at least 12 months [23]. Although a similar regimen was recommended by WHO for pulmonary TB and TBM [24], in clinical practice in China, clinicians will consider drugs with better blood–brain barrier permeation efficiency, such as isoniazid, pyrazinamide, fluoroquinolones and linezolid [25]. No study has specifically evaluated the regimens recommended by WHO or the adjusted regimens according to blood–brain barrier permeation, but the existence of drug resistant TBM may at least justify some of those activities.

The main limitations of ours and previous studies are the retrospective collection of data. Due to the scarce miliary TB, a perspective cohort requires a large number of patients that would be hard to achieve. Secondly, the time required for each examination to be performed in the episode of the disease was not comparable, which causes some bias on the sensitivity of each test. Thirdly, some patients had multiple tests for CSF biochemistry examination and bacteriological examination, and the positive rates of them were accumulated rates in this study. Therefore, if only the first outcome was included, the positive rates of them would be even lower. Besides, only two third of the enrolled patients had a sputum examination. Fourthly, all the enrolled patients were HIV-uninfected. Thus, the results did not reflect the realistic disease condition, but only HIV-uninfected ones. Finally, the decision whether to perform CSF biochemistry examination, kind of bacteriological examination, methods for neuroimaging of each patient was not according to the standard criteria, but at the discretion of the attending physician. Although, perhaps not ideal for a clinical evaluation, this represented the conditions in realistic.

## Conclusion

Almost all miliary TB had CNS involvement and MRI demonstrated outstanding potential over other methods. Therefore, a routinely screening of CNS TB should be strongly suggested in miliary TB and MRI could be used as the initial approach in resources rich settings.

## Data Availability

The datasets analysed during the current study are not publicly available due confidentiality but are available from the corresponding author on reasonable request.

## Abbreviations

TB: Tuberculosis
Mtb: *Mycobacterium tuberculosis*
CNS: Central nervous system
CSF: Cerebrospinal fluid
CT: Computed tomography
MRI: Magnetic resonance imaging
Xpert: Xpert MTB/RIF
TBM: Tuberculous meningitis
